# Ascorbic Acid and Gene Expression: Another Example of Regulation of Gene Expression by Small Molecules?

**DOI:** 10.2174/138920210790217936

**Published:** 2010-03

**Authors:** Sophie Belin, Ferdinand Kaya, Stéphane Burtey, Michel Fontes

**Affiliations:** 1 Therapy of Genetic Disorders, EA 4263, Faculté de Médecine de la Timone, Université de la Méditerranée, Marseille, France; 2 Present address: UMR 8080, Développement, Morphogenèse et Évolution, Université de Paris 11, France

## Abstract

Ascorbic acid (vitamin C, AA) has long been considered a food supplement necessary for life and for preventing scurvy. However, it has been reported that other small molecules such as retinoic acid (vitamin A) and different forms of calciferol (vitamin D) are directly involved in regulating the expression of numerous genes. These molecules bind to receptors that are differentially expressed in the embryo and are therefore crucial signalling molecules in vertebrate development. The question is: is ascorbic acid also a signalling molecule that regulates gene expression?

We therefore present and discuss recent publications that demonstrate that AA regulates the expression of a battery of genes. We offer a clue to understanding the biochemical mechanism by which AA regulates gene expression. Finally we will discuss the question of a receptor for AA and its potential involvement in embryonic development and cell differentiation.

## INTRODUCTION

In humans, L-Ascorbic acid (vitamin C) is essential for life (which is why it is called a vitamin) by virtue of its antioxidant properties, which protect cells against oxidative stress [1]. Vitamin C deficiency is termed scurvy [2]. James Lind, in 1747, was the first to conclude that eating fruits could prevent scurvy. During the 18^th^ and 19^th^ centuries, foods able to prevent scurvy were termed antiscorbutic, although the chemical principle was not identified. Albert Szent-Gyorgyi’s group first isolated the antiscorbutic principle and named it ascorbic acid (he received the Nobel Prize in 1937).

Ascorbic acid (AA) is a sugar acid with the same furanose ring as ribose. Most animals and plants synthesize AA from glucose through a four-enzyme pathway [3-5]. However, primates (as well as guinea pigs) have lost the ability to synthesize ascorbic acid because they lack one enzyme, L-gulonolactone oxidase, in the biosynthetic process [6,7]. Therefore, normal primate diets should include foods containing ascorbic acid (fruits, sauerkraut, ...).

Ascorbic acid is liberated in the digestive tract by digestion and transported into the blood by SVCT1 (SLC23A1) [8-10], a glycoprotein located on the apical and basal faces of the epithelial cells [11]. SVCT1 is also located in the kidney [12,13], where it participates in reabsorption into the circulation.

SVCT2 (SLC23A2) is a membrane protein located at the apical poles of polarized cells [14]. It transports AA into cells *via* a Na-dependent mechanism, which allows the intracellular AA concentration to exceed the extracellular concentration. AA binding to SVCT2 is very specific, as most closely-related analogues are not properly transported by it [15]. The intracellular distribution of AA and exact biological role of SVCT2 have not been fully elucidated.

The metabolic and biochemical properties of AA have been extensively documented. Most of them are associated with the chemical nature of the molecule, indicating its antioxidant properties. Surprisingly, until recently, AA was not considered a potential signalling molecule as (e.g.) vitamins A and D are. We will discuss this new hypothesis and its significance for gene expression in the following pages and document it by reference to recent publications.

## ASCORBIC ACID AND GENE EXPRESSION

As mentioned above, AA has long been considered either as an enzymatic co-factor or as a molecule that acts through the antioxidant properties attributable to its chemical structure. The first indication that it might also modulate gene expression was documented in a series of articles describing the stimulation of procollagen mRNA by ascorbate [16-18]. The authors clearly demonstrated that this effect was at the transcriptional level. Two other recent papers reported the action of AA on expression of the gene encoding a new form of collagen involved in extracellular matrix formation [19,20]. However, no clear explanation has been proposed, except for indirect effects such as hydroxyproline oxidation [21].

Therefore, although AA has been shown to regulate the expression of genes encoding extracellular matrix proteins, no additional gene or gene family was proposed until recently. In 2004, Passage *et al*. [22], demonstrated that treatment of a mouse model of the Charcot-Marie-Tooth 1A disease reverts, at least partly, to the transgenic mouse phenotype. This disease is due to the overexpression of a major myelin gene, *PMP22*, and AA treatment lowers *PMP22* expression. In the same year, using microarrays representing about 6000 genes, Shin *et al*. published a series of genes responding to AA treatment of embryonic stem cells [23]. Most of the overexpressed genes belonged to gene families involved in neurogenesis, maturation and neurotransmission. More recently, Park *et al*. published a proteomic analysis of cancer cells treated with AA [24]. The most relevant effects seem to be overexpression of RKIP and Annexin A5. Finally, in February this year, Belin *et al*. published an article describing changes of gene expression in normal and cancer cells treated with increasing doses of AA [25]. They used pangenomic arrays. The most striking result was that only 30 genes were down-regulated by AA treatment. Among these, 12 belonged to two families, tRNA synthetases and translation initiation factors, involved in cell division. The authors demonstrated that AA stops cell proliferation *in vivo* and *in* *vitro*, and kills the dividing cells by necrosis at higher concentration.

To our knowledge, only one mechanism by which AA could modulate gene expression has been proposed, through modulation of the intracellular cAMP pool; we will discuss this later. Are genes expressed and regulated through the cAMP-dependent pathway the only ones affected by AA treatment? That remains an open question.

A general question regarding the control of gene expression by small molecules concerns a potential receptor. Receptors for Vit A and Vit D have been well described and characterized (see below), but no classical receptor has been described for AA so far. However, two proteins with high affinity for AA have been described, SVCT1 and SVCT2 (for review see [26]). Both are AA transporters and act through a Na-dependent mechanism. SVCT1 is located in the intestine and kidney and is involved in AA transport into the blood. In contrast, SVCT2 is present in the plasma membranes of different cell types. It has a very specific affinity for L-ascorbic acid and does not recognize closely-related molecules [15]. It enables AA to accumulate inside cells, though we do not know exactly where the intracellular AA is located.

Interestingly, the gene encoding a key enzyme in AA biosynthesis, L-gulono-gamma-lactone synthase, accumulates mutations in man and guinea pig [27, 28]. In consequence, these two species cannot synthesis AA. In contrast, SVCT2 is one of the most evolutionarily conserved molecules and no species lacks this key protein. We may conclude that it is not AA biosynthesis itself, but the accumulation of AA in cells mediated by SVCT2, that is most important for organisms. The involvement of SVCT2 in AA signal reception will thus be very interesting to investigate.

## GENE EXPRESSION AND BIOLOGICAL/BIOCHEMICAL FUNCTIONS OF ASCORBIC ACID

What could be the mechanism by which AA acts on gene expression?

The first clearly established function of AA was to prevent scurvy. Evidence that AA was necessary to prevent the appearance of this fatal disease was described above, with no indication of other roles this molecule might play.

From its chemical structure, AA is an electron donor and therefore a reducing agent. It thus has two different biochemical roles: as an antioxidant and as an enzymatic cofactor. It is an electron donor for different enzymes, including three involved in collagen hydroxylation [21, 29], two in carnitine synthesis [30,31], and others in norepinephrin [32] and tyrosine [33] synthesis. AA deficiency is therefore associated with extracellular matrix defects that are probably implicated in the vascular problems observed in scurvy.

This enzymatic property certainly has an impact on cell metabolism that could influence gene expression. However, these mechanisms are probably not directly involved in the control of gene expression and cell signalling. The first explanation came from recent results obtained by our group. As we described above, AA treatment lowers the expression of *PMP22*, which is overexpressed in CMT1A patients [34]. This is probably the basis of the phenotypic correction observed in a CMT animal model. Expression of *PMP22* is under the control of cAMP, *via* fixation of CREB on a site located on the promoter (1.5 kb from the transcription initiation site) [34-37]. Incubation of Schwann cell lines with increasing concentrations of AA results in dosage-dependent inhibition of *PMP22* expression. In addition, the intracellular cAMP pool is decreased when cells are incubated with increasing AA concentrations [34]. This inhibition is specific to AA and does not occur with other antioxidants [38]. Recently, using classical enzymatic experiments, it has been demonstrated that AA is a competitive inhibitor of adenylate cyclase [39], probably because AA and ATP both have a furanose ribose ring (with different side chains). Therefore, AA could repress the expression of genes under the control of the cAMP-dependent pathway.

What could be the effect of this new property on biological phenomena? Two lines of research indicate that AA may be involved in cell differentiation. The first regards Schwann cell differentiation and myelination. It has been demonstrated that AA is needed in Schwann cell/axon co-cultures to induce myelin formation [40-42]. However, no clear mechanism has been demonstrated other than antioxidant properties. The second line of research has been initiated more recently and involves embryonic stem cell (ES) differentiation. Using high throughput screening of a chemical library, Takahashi *et al*. found that AA was the only molecule present in the library that promoted differentiation of murine ES in cardiomyocytes [43]. They also demonstrated that this effect is specific to AA and is not shared with other antioxidants, so antioxidant mechanisms are probably not involved in this process.

Finally, Sotiriou *et al*. described the phenotype of a mouse mutant in which the gene encoding SVCT2 was inactive [44]. In homozygotes, the AA concentration in the extracellular fluid was normal but the molecule could not enter cells. Embryos died in the perinatal period with anomalies in the lungs and blood vessels.

These different observations clearly raise questions about AA as a signalling molecule in mammalian development and differentiation. These are fascinating new topics that will probably develop in the near future.

## OTHER VITAMINS AND GENE EXPRESSION

As already mentioned, several "vitamins" have been shown to play roles in the regulation of gene expression. We will focus on the two best-documented molecules: Vitamin A and Vitamin D.

### Vitamin A

Vitamin A and its bioactive metabolites have pleiotropic effects in all tissues involving cellular development, proliferation, differentiation, metabolism and apoptosis.

About 50% of the vitamin A intake is derived from animal products that contain retinol and retinyl esters; the remaining 50% is provided by plant carotenoids. The active form of vitamin A is retinoic acid (RA). The amount of vitamin A needed by an adult is 5-600 mg per day, and any prolonged deficit or excess in this daily intake causes serious heath problems. Vitamin A deficiency (VAD) is deleterious to embryo formation (reviewed in [45]) and is responsible for a large array of congenital malformations affecting the ocular, cardiac, respiratory and urogenital systems. Conversely, retinoids administered in excess to pregnant females typically induce truncation of the anterior brain (an- or ex-encephaly) and posteriorization of hindbrain rhombomeres. So, in embryos, vitamin A excess or deficit leads to severe anomalies of development. The function of RA signalling in development has been extensively studied using transgenic mice [46]. The main conclusion is that signalling through RARs is indispensable for embryonic patterning and organogenesis, and the RA signalling pathway plays a crucial role in development. The level of RA is critical. In adults, VAD is associated with xerophthalmia (irreversible degradation of the cornea) leading in the worst case to blindness. The critical role of vitamin A in maintaining the immune response to infectious diseases is well documented. Characteristic adverse effects of hypervitaminosis A are alopecia, elevation of serum triglycerides, hyperostosis, and extraskeletal calcification.

The widespread nature of retionoid bioactivities is due, in large part, to their ability to regulate the expression of target genes. All-*trans* and 9-*cis* retinoic acid, the retinoids active in regulating gene expression in cell nuclei, are ligands that bind and activate cognate retinoid receptors. These receptors, in turn, function as transcription factors that regulate the expression of target genes.

In the nucleus there are two distinct classes of retinoid receptors, the retinoic acid receptors (RAR) and retinoid X receptors (RXR), and the nonsteroid, thyroid/retinoid/vitamin D superfamily of nuclear receptors (NRs). These are Class II NRs, which heterodimerize with RXR and bind to direct repeat (DR) elements in the target genes [47].

The classical retinoid response element of a target gene is a DR of the motif 5V-PuG(G/T)TCA-3V spaced by 1, 2 or 5 base pairs (DR1, DR2 and DR5, respectively) [48-50]). It has been established that RARs are ligand-dependent transcription factors, and repress transcription in the absence of ligand.

Transcriptional activation by NRs entails a complex mechanism (for review see [51]). Briefly, the first step is the binding of the ligand to RAR or RXR, leading to conformational changes in the NRs. The second critical step is DNA binding. The third is the recruitment of coactivators or the release of corepressors (numerous functional groups of coactivators and corepressors have been characterized). The last step leads to a modified chromatin structure and finally to an increased transcription initiation rate.

Beside activation *via* NRs, RA signalling could lead to transrepression, responsive to repression of transcription factors such as AP1 and NF-kB.

The mechanisms of gene regulation by RA have been reviewed using *PEPCK* as a model gene; it exemplifies the pleiotropic effects of retinoids [52].

A listing and categorization of retinoid-responsive genes cited 532 RA-target genes. This review distinguished genes subject to direct, classic retinoid signalling from those subject to indirect RA regulation [53]. Category 3 included 27 genes unquestionably controlled through the classical RA pathway in some cellular context(s): *ADH1C, CD38, CDX1, CEBPE, CRABP2, CRYAB B, DRD2, EGR1, ETS1, FOXA1, H1F0, HOXA1, HOXA4, HOXB1, HOXB4, HOXD4, HSD17B1, IL2RA, PCK1, PIT1, RARA, RARB, RARG, RBP1, SFTPB, TGM2, UCP1.* Category 2 included 105 genes that may be modulated at the transcriptional level, but other indicators of direct regulation have not yet been explored. In most cases, there is still a lack of data relating to response elements or RAR.RXR involvement. Category 1 included 267 genes. RA regulates them in some way; there are insufficient data to distinguish between direct or indirect regulation. Category 0 included 124 genes that seem to be regulated indirectly in the contexts studied.

These vast arrays of genes regulated by RA signalling, and the developmental effects of RA signalling, make this small molecule more than a vitamin but a real morphogen.

### Vitamin D

Vitamin D (vitD) is a key vitamin in the control of calcium level, which is the most tightly-regulated plasma ion level. VitD coordinates the participation of four different tissues in calcium homeostasis - kidney, intestine, bone and parathyroid - so it is clearly a hormone more than a vitamin. It is essential for the development and maintenance of a mineralized skeleton by controlling bone formation and also the absorption of calcium and phosphate, two keys components of bone, by the intestine. It is not only involved in the regulation of calcium homeostasis and bone mineralization *via* vitamin D receptor (VDR) activation but also has anti-proliferative, pro-differentiation, and immunomodulatory activities. The vitD endocrine system also participates in the regulation of blood pressure, volume homeostasis, cardiac function, and protection of renal cellular integrity.

Humans obtain vitamin D from exposure to sunlight, from their diet, and from dietary supplements. A diet high in oily fish prevents vitD deficiency. Solar ultraviolet B radiation (wavelength 290-315 nm) penetrates the skin and induces the photolytic conversion of 7-dehydrocholesterol to previtamin D3, followed by thermal isomerization to vitamin D3. The first step in the metabolic activation of vitD is hydroxylation of carbon 25, which occurs primarily in the liver and is catalyzed by 25-hydroxylase. 25-OH-D3 is the metabolite measured to evaluate vitamin D deficiency. The second step in vitD bioactivation is the formation of 1,25-dihydroxyvitamin D3 [1,25-(OH)2D] from 25-hydroxyvitamin D3. This occurs under physiological conditions, mainly in the kidney. Renal 1-hydroxylase activity is highly regulated, in keeping with the potent activity of its product in calcium homeostasis. Dietary calcium regulates the enzyme, directly through changes in serum calcium, and indirectly by altering parathyroid hormone (PTH) levels [53].

VitD deficiency (VDD) is associated with rickets in children and osteomalacia in adults [54]. Besides this well-described bone effect, VDD is suspected of being associated with numerous diseases: muscle weakness and falling, cancer, cardiovascular diseases and autoimmune diseases (see review in [55]).

Excess vitD is associated with hypercalcemia and hypercalciuria and can lead to serious complications. It may be secondary to excessive ingestion of vitamin or to excessive and unregulated production of 1,25-OH2-D3, by granulomata in sarcoidosis or tuberculosis for example.

The clinical effects of deficiency or excess in vitD are directly linked to the regulation of gene expression by active vitD metabolites *via* nuclear receptors. 1,25(OH)2D3 regulates gene expression in target cells by binding to the vitamin D receptor (VDR). The liganded VDR heterodimerizes with the retinoid X receptor (RXR), binds to vitamin D response elements (VDREs) in the promoters of target genes and, together with coactivators or corepressors, affects target gene transcription. Directly or indirectly, 1,25-OH2 D 3 controls between 200 and 500 of the 20,488 genes in the human genome, including genes responsible for regulating cellular proliferation, differentiation, apoptosis, and angiogenesis.

Most of the biological activities of 1,25(OH)2D3 require a high-affinity receptor, the VDR, an ancient member of the NR II superfamily. Like the other members of the steroid receptor family, the VDR acts as a ligand-activated transcription factor.

We may summarize five major steps in the control of gene transcription by the VDR:

- Ligand binding (in the cytoplasm). In the absence of ligand, most of the VDR is present in the cytoplasm. Ligand binding induces conformational changes, enhancing dimerization with RXR.
- Heterodimerization with the retinoid X receptor (RXR). RXR, a nuclear receptor for 9-*cis* retinoic acid, is an obligatory partner of VDR in mediating 1,25-(OH)2D3 action.
- Translocation of the heterodimer VDR-RXR with ligand from the cytoplasm to the nucleus.
- Binding of the heterodimer to vitD response elements (VDREs) in the promoters of 1,25(OH)2D-responsive genes. Canonical VDREs are direct repeats of 5-AGG/TTCA-3, or a minor variation of this motif, separated by three nucleotides and commonly referred to as DR-3 motifs.
- Recruitment of VDR-interacting nuclear proteins (coregulators) into the transcriptional preinitiation complex, which markedly enhance or suppress the rate of gene transcription by the VDR. Ligand binding in general increases the affinity of the VDR for various protein cofactors that act as a bridge between the RXR-VDR heterodimer and the basal Pol II transcription machinery. In transcriptional repression by the VDR, such as that of *PTH*, binding of the VDR-RXR complex to a negative VDRE recruits corepressors of the family of histone deacetylases (see review in [56]).

The VDR protein is modular in nature and like other nuclear receptors can be divided into three regions with well-characterized functions. The NH_2_-terminal region contains a ligand-independent transactivation function, activation function-1 (AF-1). The central region contains the DNA binding domain consisting of two C2-C2 type zinc fingers, which target the receptor to VDREs. The C-terminal region contains a multifunctional domain harbouring the ligand binding domain (LBD), the RXR heterodimerization motif, and a ligand-dependent transactivation function, AF-2.

The VDR can modulate the expression of vitD-responsive genes in three different ways. (1) The expression of certain genes is positively regulated by VDR binding to the VDREs in their promoter regions. These genes include osteocalcin, osteopontin, receptor activator of NF-κB ligand (RANKL), and carbonic anhydrase II, which are involved in extracellular bone matrix formation and bone remodelling. Other genes are the cell adhesion molecule 3 integrin, tumor suppressor p21, calbindin-9k, 24-hydroxylase, CYP3A4, involucrin, phospholipase C (PLC) 1, and IGF binding protein (IGFBP)-3. (2) The expression of other genes is negatively regulated by VDR binding to negative VDREs. The best-described genes that are down-regulated by VDR signalling are PTH and 1-hydroxylase, which are involved in mineral homeostasis. Other genes such as those associated with cytokines, IL-2, IL-12, TNF, interferon and granulocyte-macrophage colony-stimulating factor, and proliferation-associated genes such as those for epidermal growth factor receptor, c-myc and K16, are repressed by VDR ligands. (3) The expression of some genes is inhibited by antagonizing the action of certain transcription factors such as nuclear factor (NF)-AT and NF-kB.

In cancer cells, numerous genes regulated by the VDR have been identified by expression microarrays. Many of them are involved in the cell cycle. For example, the VDR induces the expression of the p21 and p27 genes and plays a role in the arrest of cellular proliferation and cancer control (see review in [57]).

In the cardiovascular system, 1,25-OH-D3 controls the expression of key genes involved in blood pressure control such as those for renin and angiotensin II. VitD analogues also control genes involved in the progression of glomerular disease in diabetes. In association with an RAS inhibitor, a VDR agonist suppresses the induction of fibronectin, TGF-β and MCP-1 and reverses the decline of the slit diaphragm proteins nephrin, Neph-1, ZO-1 and actinin-4. Combination therapy with an AT1 blocker and a vitD analogue markedly ameliorates diabetic nephropathy. VitD plays an important role in the immune system. The VDR is present in activated human mononuclear leukocytes and in B and T lymphocytes. In the adaptive immune system, 1,25-dihydroxyvitamin D3 plays an immunoregulatory role. Toll-like receptor activation of human macrophages up-regulates expression of the VDR and the 25(OH)D3-1 hydroxylase genes; induction of cathelicidin, an antimicrobial peptide, through VDR-mediated gene expression enables intracellular *Mycobacterium tuberculosis* to be killed.

In the endocrine system, besides the parathyroid, vit D acts on pancreatic cells; 1,25-OH2-D3 induces insulin secretion, and 25(OH)D concentration correlates positively with insulin sensitivity.

Because of their antiproliferative effects, Vit D analogues could be a major new drug family for cancer treatment (see review in [58]).

Vitamins A and D are thus good examples of small molecules that are more than simple nutritional complements. Vit A plays a critical role in organ development and morphogenesis. Vit D and its analogues are potentially important drugs with multiple targets - cancer, hypertension and cardiovascular diseases – because of its pleiotropic effects on gene transcription. We believe that a similar picture is emerging for vitamin C. Vits A and D act by specific binding to a receptor that is translocated to the nucleus and regulates gene expression. AA seems to act differently. We know of no transcriptional factor that binds AA and regulates gene expression. Regarding the question of a receptor, the situation is more complex. An important question is whether we may consider the transporter SVCT2 as a receptor. Regarding its specificity for AA the answer is yes, but it is not a nuclear receptor, and regulation of gene expression by the intracellular concentration of AA is probably not direct.

## CONCLUSION

AA has long been studied by groups involved in nutrition and has been considered a food supplement. The major biochemical function proposed for AA has been its antioxidant property and the major cellular function has been to promote extracellular matrix formation by stabilising collagen structure.

However, although these activities do occur *in vivo*, recent progress allows us to consider additional functions, among which embryonic/cell differentiation is probably the most promising. It will be very interesting to follow the AA distribution in embryos and to try to link it to embryonic signalling. The action of AA on cell division is probably a good route. We already know that the intracellular AA concentration is heterogeneous in adults [59]; some tissues have high concentrations, others have much lower ones. If the same is true in embryos, we may assume that the division rate could be affected by the local AA concentration. As the progression and inhibition of cell division are crucial during embryogenesis and cell differentiation, we could hypothesize that the local AA concentration influences these processes. This heterogeneity in AA concentration is probably linked to the concentration of the transporter SVCT2 [10], which may act as both a transporter and a receptor. The local AA concentration could modulate the expression of a battery of genes and influence cell division. An alternative mechanism may be direct action of AA on the expression of genes involved in mammalian development.

Regarding the signalling pathway that could be involved, we suggest cAMP pathways (probably through CREB synthesis, as seems to be the case for *PMP22*). As our recent work suggests, AA is a competitor of adenylate cyclase activity, suggesting that it is a global regulator of the intracellular cAMP pool. Therefore, AA could modulate the expression of a battery of genes expressed under the control of cAMP-dependent pathways. Some have already been described but a systematic evaluation of genes that could be modulated during development will be interesting.

A final question concerns the evolution of this mechanism and this new property of AA. Could we extrapolate our hypothesis beyond mammals? This is a fascinating question and currently there is no clear answer. However, we note that the transporter SVCT2 is strongly conserved through evolution. Human and Drosophila SVCT2s show conservation of about 70% of the coding sequence. If this extends to conservation of function, AA may be one of the oldest developmental signalling molecules.
